# Oncological Response and Predictive Biomarkers for the Checkpoint Inhibitors in Castration-Resistant Metastatic Prostate Cancer: A Systematic Review and Meta-Analysis

**DOI:** 10.3390/jpm12010008

**Published:** 2021-12-23

**Authors:** Omar Fahmy, Nabil A. Alhakamy, Mohd G. Khairul-Asri, Osama A. A. Ahmed, Usama A. Fahmy, Claudia G. Fresta, Giuseppe Caruso

**Affiliations:** 1 Department of Urology, University Putra Malaysia, Seri Kembangan 43400, Malaysia; omarfahmy.ahmed@upm.edu.my (O.F.); khairulasri@upm.edu.my (M.G.K.-A.); 2 Department of Pharmaceutics, Faculty of Pharmacy, King Abdulaziz University, Jeddah 21589, Saudi Arabia; nalhakamy@kau.edu.sa (N.A.A.); oaahmed@kau.edu.sa (O.A.A.A.); uahmedkauedu.sa@kau.edu.sa (U.A.F.); 3 Advanced Drug Delivery Research Group, Faculty of Pharmacy, King Abdulaziz University, Jeddah 21589, Saudi Arabia; 4 Center of Excellence for Drug Research and Pharmaceutical Industries, King Abdulaziz University, Jeddah 21589, Saudi Arabia; 5 Mohamed Saeed Tamer Chair for Pharmaceutical Industries, King Abdulaziz University, Jeddah 21589, Saudi Arabia; 6 Department of Biomedical and Biotechnological Sciences, University of Catania, 95125 Catania, Italy; forclaudiafresta@gmail.com; 7 Department of Drug and Health Sciences, University of Catania, 95125 Catania, Italy

**Keywords:** prostate cancer, immune checkpoint inhibitors, immunotherapy, metastatic castration-resistant prostate cancer

## Abstract

Recently, checkpoint inhibitors have been investigated in metastatic prostate cancer, however their overall effect is unclear and needs to be further investigated. Objectives: The aim of this systematic review is to investigate the oncological response of metastatic castration-resistant prostate cancer patients to immune checkpoint inhibitors. Methods: Based on the preferred reporting items for systematic reviews and meta-analyses (PRISMA) statement, a systematic review of the literature was conducted through online electronic databases and the American Society of Clinical Oncology (ASCO) Meeting Library. Eligible publications were selected after a staged screening and selection process. RevMan 5.4 software was employed to run the quantitative analysis and forest plots. Risk of bias assessment was conducted using the Cochrane tool and Newcastle–Ottawa Scale for the randomized and non-randomized trials, respectively. Results: From the 831 results retrieved, 8 studies including 2768 patients were included. There was no significant effect on overall survival (OS) (overall response (OR) = 0.98; Z = 0.42; *p* = 0.67). Meanwhile, progression-free survival (PFS) was significantly better with immune checkpoint inhibitors administration (OR = 0.85; Z = 3.9; *p* < 0.0001). The subgroup analysis for oncological outcomes based on programmed death ligand 1 (PD-L1) positivity status displayed no significant effect, except on prostate-specific antigen response rate (PSA RR) (OR = 3.25; Z = 2.29; *p* = 0.02). Based on DNA damage repair (DDR), positive patients had a significantly better PFS and a trend towards better OS and overall response rate (ORR); the ORR was 40% in positive patients compared to 20% in the negative patients (OR = 2.46; Z = 1.3; *p* = 0.19), while PSA RR was 23.5% compared to 14.3% (OR = 1.88; Z = 0.88; *p* = 0.38). Better PFS was clearly associated with DDR positivity (OR = 0.70; Z = 2.48; *p* = 0.01) with a trend towards better OS in DDR positive patients (OR = 0.71; Z = 1.38; *p* = 0.17). Based on tumor mutation burden (TMB), ORR was 46.7% with high TMB versus 8.8% in patients with low TMB (OR = 11.88; Z = 3.0; *p* = 0.003). Conclusions: Checkpoint inhibitors provide modest oncological advantages in metastatic castration-resistant prostate cancer. There are currently no good predictive indicators that indicate a greater response in some patients.

## 1. Introduction

Prostate cancer was the second most frequent cancer and the fifth largest cause of cancer-related deaths among males globally in 2020 [1]. Therapeutic options for advanced prostate cancer have expanded in the last few years as a result of a better knowledge of the molecular processes that underpin metastatic spread, particularly the crucial involvement of the tumor microenvironment [2]. Prostate cancer is incurable once it has spread to other parts of the body [3]. The long-standing standard of therapy for metastatic prostate cancer is androgen deprivation [4]. Aside from androgen deprivation therapy (ADT), which is the cornerstone of metastatic prostate cancer care, therapeutic options mostly consist of either innovative hormonal therapies (abiraterone, enzalutamide, and apalutamide) or taxane-based chemotherapy (docetaxel and cabazitaxel) [5,6]. Castrate-resistant prostate cancer (CRPC) develops when tumors cease responding to androgen deprivation. Docetaxel, cabazitaxel, abiraterone, enzalutamide, sipuleucel-T, and the bone-specific radionuclide radium-223 represent all therapy possibilities for individuals with metastatic CRPC (mCRPC). These treatments are not curative and may have a low tolerability [7].

Given the limited therapy choices for the vast majority of patients and the promising results of immune checkpoint inhibitors (ICIs) in other advanced diseases such as melanoma and lung cancer, there is a growing emphasis on treating prostate cancer with ICIs [8,9]. Although immune checkpoint blockade has been shown to be effective in urothelial and renal-cell carcinomas [10,11], prostate cancer has a more immunosuppressive microenvironment than these other genitourinary malignancies, suggesting that mCRPC may be less susceptible to immune checkpoint blockade [12,13].

The aim of this systematic review is to investigate the oncological response of mCRPC to ICIs. In addition, we explored several potential predictive biomarkers that could help in patient selection for future trials to maximize the benefit coming from drug treatment.

## 2. Materials and Methods

### 2.1. Search Strategy

Following the preferred reporting items for systematic reviews and meta-analyses (PRISMA) criteria [14,15], we conducted online systemic search through online electronic databases (PubMed, EMBASE, Wiley Online Library, and Cochrane databases). The following keywords were utilized during the search: metastatic prostate cancer; immunotherapy; checkpoint inhibitors; castration-resistant. Exclusion criteria were: (1) review articles; (2) case reports; (3) letters to editors and editorial comments; (4) repeated publications; (5) non-controlled trials without subgroup analysis for the oncological outcomes; (6) non-English articles; (7) trials on other prostate cancer cohorts rather than mCRPC. All results initially assessed by the title, with or without abstract assessment, were followed by full-text assessment. Eventually, we included the controlled trials reported on checkpoint inhibitors in mCRPC, or non-controlled trials but with subgroup analysis and comparisons.

### 2.2. Data Extraction

Data was independently extracted by two authors and checked by a third one: total number of patients, trial number, type and status, patients’ criteria, investigated drugs, overall response rates (ORR), prostate-specific antigen response rate (PSA RR), overall survival (OS), progression free survival (PFS), follow-up duration. ORR was defined as number of patients with partial or complete response to treatment according to the response evaluation criteria in solid tumors (RECIST) 1.1 criteria [16]. PSA RR was defined as reduction of PSA by at least 50% from the baseline. Both previous definitions were used in all the included studies. Subgroups’ oncological outcomes were based on the investigated biomarkers such as programmed death ligand 1 (PD-L1) status, DNA damage repair (DDR) status, and tumor mutation burden (TMB). Dichotomous data were extracted as number of events and total numbers. Survival data were extracted as hazard ratio (HR) and 95% confidence interval (CI). When HR and CI were not reported, Tierney’s method was employed to estimate the HR and CI from Kaplan–Meier curves [17].

### 2.3. Primary Outcomes

The primary outcome of this systematic review and meta-analysis was to investigate the oncological response of mCRPC to checkpoint inhibitors and the biomarkers associated with a better response. 

### 2.4. Statistical Analysis

The Nordic Cochrane Centre (Cochrane Collaboration, Copenhagen) employed Review Manager (RevMan) software version 5.4 (Cochrane library, London, UK)) for statistical analysis and the creation of forest plots for this meta-analysis. For time to event data (OS, PFS) we calculated the OR with 95% CI using Log HR and standard error (SE). For dichotomous data (ORR, PSA RR) we calculated the OR with 95% CI using number of events and total numbers. The I^2^ value was used to determine the heterogeneity of the research. For I^2^ < 50% fixed effect model was used, while in the case of I^2^ ≥ 50% random effect model was examined. The Z-test was used to assess the overall impact. *p*-values < 0.05 were considered significant in all tests [18].

### 2.5. Risk of Bias Assessment

For randomized trials, Cochrane bias assessment module of RevMan 5.4 software was employed. For non-randomized trials, Newcastle–Ottawa Scale was employed and scores of 7–9, 4–6, and 4 were classified as having a low, moderate, or high risk of bias, respectively (http://www.ohri.ca/programs/clinical_epidemiology/oxford.asp (accessed on 5 October 2021)).

## 3. Results

### 3.1. Search Results

**Figure 1:**
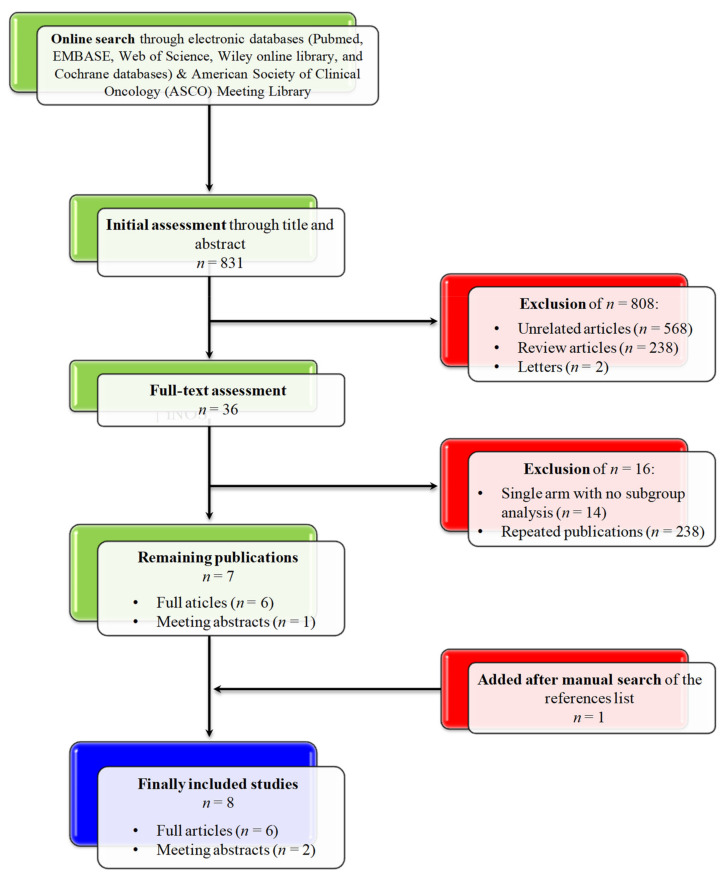
CONSORT diagram for the screening and selection processes of the included studies.

Initial search in electronic databases revealed 831 results which underwent initial assessment. Twenty three publications underwent full-text assessment, and finally, eight publications (six journal papers [19,20,21,22,23,24], and two meeting abstracts [25,26]) were considered. A total number of 2768 patients with mCRPC were included in the pooled analyses, after exclusion of one cohort (*n* = 59 patients) from one study not eligible for subgroup analysis [21]. The flow of screening and selection process is showed in Figure 1, while the summary of the included studies is provided in Table 1.

**Figure 2:**
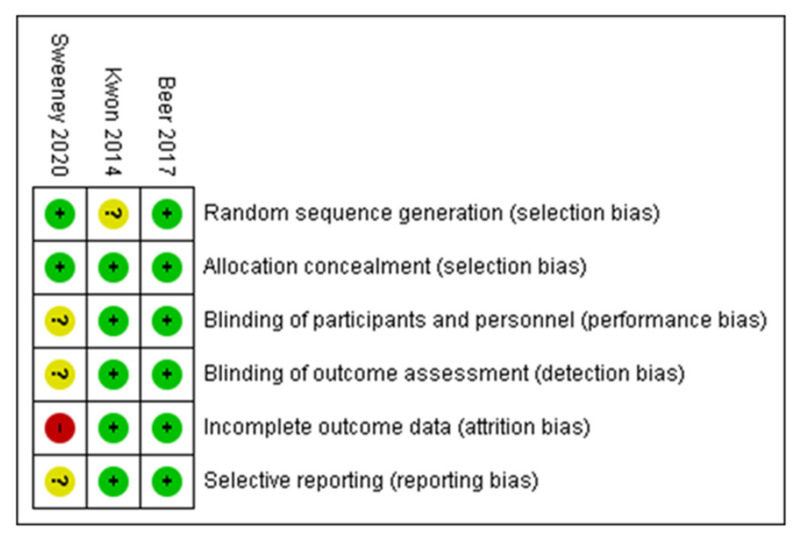
Risk of bias in the randomized trials (green = low risk, yellow = unclear, red = high risk). Sweeney et al., 2020 [25], Kwon et al., 2014 [24], Beer et al., 2017 [23].

Risk of bias assessment is available in Table 2 for the five non-randomized trials [19,20,21,22,26], and in Figure 2 for the three randomized trials [23,24,25].

### 3.2. OS and PFS

**Figure 3:**
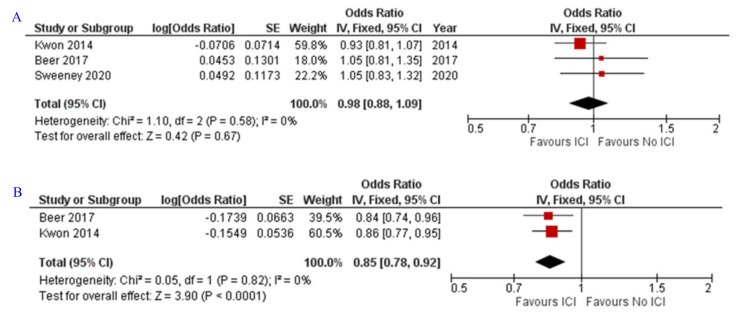
Forest plot for the impact of ICIs, on the (**A**) OS and (**B**) PFS. ICIs, immune checkpoint inhibitors; OS, overall survival; PFS, progression-free survival. Sweeney et al., 2020 [25], Kwon et al., 2014 [24], Beer et al., 2017 [23].

The analysis of three randomized phase III studies including 2395 patients showed no significant effect of ICIs in improving the OS (OR = 0.98; Z = 0.42; *p* = 0.67) [23,24,25] (Figure 3A). Meanwhile, PFS form two randomized phase III studies with 1636 patients was significantly better with ICIs administration (OR = 0.85; Z = 3.9; *p* < 0.0001) [23,24] (Figure 3B).

### 3.3. Impact of PD-L1 Status on Oncological Response

**Figure 4:**
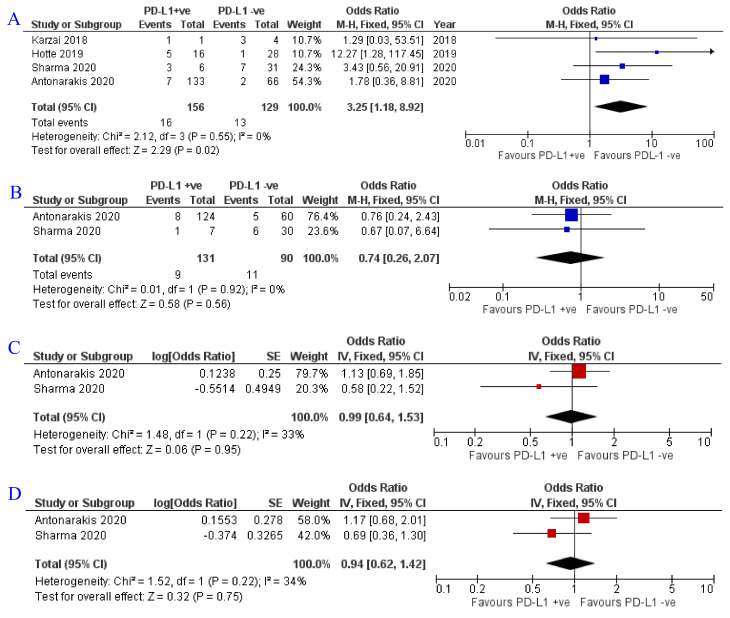
Forest plots for the impact of PD-L1 status on (**A**) ORR, (**B**) PSA RR, (**C**) PFS, and (**D**) OS. PD- L1, programmed death ligand 1; ORR, overall response rate, PSA RR, prostate-specific antigen response rate. Antonarakis et al., 2020 [21], Sharma et al., 2020 [19], Hotte et al., 2019 [26], Karzai et al., 2018 [22].

Subgroup analysis for oncological outcomes based on PD-L1 positivity status was feasible for ORR, PSA RR, OS, and PFS. There was no significant effect, except on ORR; PSA RR was 6.9% (9/131) in PD-L1 positive patients compared to 12.2% (11/90) in negative patients (OR = 0.74; Z = 0.58; *p* = 0.56) [19,21]. For ORR, despite a comparable rate 10.3% (16/156) and 10.0% (13/129) in positive and negative, respectively, yet the pooled effect displayed significant association between the PD-L1 positivity and the ORR (OR = 3.25; Z = 2.29; *p* = 0.02) [19,21,22,26]. There was almost no impact on PFS (OR = 0.99; Z = 0.06; *p* = 0.95) [19,21] or OS (OR = 0.94; Z = 0.32; *p* = 0.75) [19,21] (Figure 4).

### 3.4. Impact of DDR Status on Oncological Response

**Figure 5:**
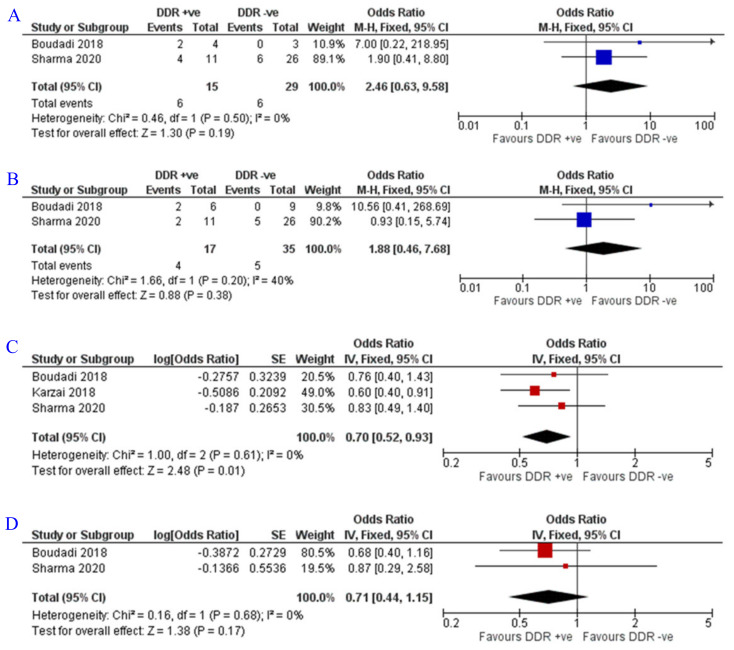
Forest plots for the impact of DDR status on (**A**) ORR, (**B**) PSA RR, (**C**) PFS, and (**D**) OS. DDR, DNA damage repair. Sharma et al., 2020 [19], Boudadi et al., 2018 [20], Karzai et al., 2018 [22].

A similar subgroup analysis was conducted based on DDR positivity. Positive patients had a significantly better PFS and a trend towards better OS and ORR; the ORR was 40% (6/15) in positive patients compared to 20% (6/29) in the negative patients (OR = 2.46; Z = 1.3; *p* = 0.19) [19,20]. PSA RR was 23.5% (4/17) compared to 14.3% (5/35), however not significant (OR = 1.88; Z = 0.88; *p* = 0.38) [19,20]. In three studies including 122 patients, better PFS was clearly associated with DDR positivity (OR = 0.70; Z = 2.48; *p* = 0.01) [19,20,22]. With regard to OS, two studies with 105 patients displayed a trend towards better OS in DDR positive patients (OR = 0.71; Z = 1.38; *p* = 0.17) [19,20] (Figure 5).

### 3.5. Impact of TMB on Oncological Response

**Figure 6:**
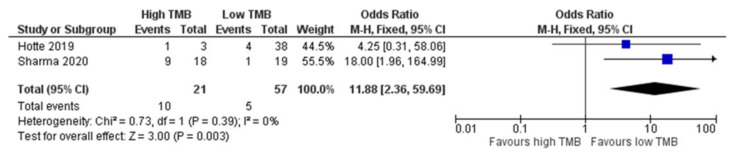
Forest plots for the impact of TMB on ORR. TMB, tumor mutation burden. Sharma et al., 2020 [19], Hotte et al., 2019 [26].

Subgroup analysis based on TMB was only feasible for ORR. Higher ORR was clearly associated with higher TMB. In a total of 78 patients from two studies, the ORR was 46.7% (10/21) with high TMB compared to 8.8% (5/57) in patients with low TMB (OR = 11.88; Z = 3.0; *p* = 0.003) [19,26] (Figure 6).

## 4. Discussion

About 6% of prostate cancer patients have metastatic disease at first time of diagnosis with about 30% 5-year relative survival rate [27]. In addition, a few patients can develop metastasis many years after receiving radiotherapy or radical prostatectomy [28]. Currently, treatment of mCRPC is a very dynamic research focus area at both preclinical and clinical levels. Despite the wide variation of available options, there is no effective medication compared to the others [7]. Furthermore, incomplete understanding of the resistance mechanism to ADT makes mCRPC therapy very challenging [2].

In the past decade, ICIs displayed a durable response up to complete regression of metastatic lesions in different types of cancers such as melanoma and non-small cell lung carcinoma [29]. ICI agents have been investigated in urological cancers such as renal cell carcinoma, urothelial carcinoma, and prostate cancer, however their benefits in prostate cancer still need to be clarified [30]. Compared to melanoma, prostate cancer is immunologically cold with about 10 times less TMB. In addition, the hypoxic environment in the prostate is less attractive for infiltration by immune cells [31,32]. Therefore, this systematic review aimed to clarify the benefits of ICIs in mCRPC.

ICIs have been investigated as monotherapy as well as in different combination approaches, such as with chemotherapy or other ICI agents [22,24,26]. Chemotherapy might increase the tumor antigenicity, alter immune-suppressive pathways, and boost effector T cell responses. This points to the possibility of better outcomes when combining immunotherapy and anticancer agents [33,34]. The combination of cytotoxic T-lymphocyte antigen 4 (CTLA-4) and programmed death-1 (PD-1) blockade has been linked to better antitumor responses; in this context, it has been shown that ipilimumab, a monoclonal anti-CTLA4 antibody, increases tumor-infiltrating T cells and up-regulates the PD-1/PD-L1 inhibitory pathway in a compensatory fashion, implying that combination therapy may be very effective [35].

Survival benefits in unselected mCRPC patients seem to be modest. Our analysis displayed no benefit for OS, however ICIs might, at least, stabilize the disease, since the PFS was significantly better in patients treated with ICIs. The pleasant outcome is when there is prolongation of both OS and PFS, however the relationship between OS and PFS is not always directly correlated. Many factors such as the type of the disease and patients’ age and performance status can affect the direction of this relationship [36]. Another explanation is that the tumor microenvironment plays a fundamental role in response to immunotherapy. This microenvironment can be heterogeneous among prostate cancer patients, possibly leading to different degrees of response to checkpoint inhibitors [37]. These results must be interpreted cautiously due to the very limited number of studies involved and the heterogeneity in protocols and cohorts.

One of the challenging points regarding ICIs is the identification of real predicative biomarkers that can help in patient selection before starting the treatment. The identification of biomarkers is usually obtained from tissue biopsies that might not be available for every patient. In addition, long time preservation and technical processing might alter the molecular properties of the tissue samples [38]. PD-1/L1 expression has been investigated at an early stage in different types of cancers for correlation with response to ICIs. A PD-L1 immunohistochemistry test has been approved by the Food and Drug Administration (FDA) as a pretreatment diagnostic test, even though PD-L1 expression patterns are not a strong predictive biomarker for identifying other cancer types [39]. PD-1/L1 expression in prostate cancer tissues has been studied in a number of preclinical investigations and the results displayed that PD-L1 expression in tumors is variable and may rise as the tumor progresses [40]. In addition, radiotherapy can influence PD-L1 expression [41]. Therefore, patient selection for ICIs based on PD-1/L1 expression only might not represent an effective solution. In clinical studies, our pooled analysis displayed better ORR but no survival or PSA reduction benefits. Patients with partial response were considered responders according to the definition used by the included studies. Those patients might have a temporary response that was followed by disease relapse. Therefore, the better ORR observed does not change the conclusion of our study. Although we already answered this question, the results were based on different patient cohorts that underwent different treatment protocols.

DDR and TMB might be more reliable than PD-L expression as potential future predictive biomarkers. It is now recognized that somatic mutations impacting DNA repair genes are present in roughly 20–25 % of metastatic prostate cancer patients [42]. Recent studies suggest that DNA damage can enhance the type I interferons (IFNs) system through the activation of the stimulator of interferon genes (STING) pathway. This, in turn, stimulates type I IFNs and cytosolic DNA sensors such as cyclic GMP–AMP synthase (cGAS), both of which effectively excite antitumor T cells [43,44]. Furthermore, concomitant genomic alterations such as homologous recombination deficiency (BRCA2, ATM, CDK12 mutations), can increase ICIs responsiveness by increasing TMB and neoantigen expression [45,46]. In addition, increased TMB might be associated with higher DDR, based on a study including 4129 prostate cancer patients [47]. In our results, DDR positivity was associated with significantly better PFS and a trend towards better OS. TMB was also associated with much higher ORR. Despite the limitations of our analysis, these results correlate with the previously mentioned thoughts regarding the possible role of the genetic-based response of mCRPC to ICIs.

The results of this systematic review and quantitative analysis are greatly limited by fundamental issues. For instance, the limited number of included studies, the number of patients, as well as the number of responders. Some studies are characterized by lack of randomization or control arm and incomplete reports. Additionally, different patients’ inclusion criteria and previous exposure to different treatments such as radiotherapy, chemotherapy, and novel ADT agents were considered. Furthermore, subgroup analysis protocols were heterogeneous among studies. The short follow-up durations should also be taken into account.

In view of the current status of ICIs in mCRPC, genetic-based selection and profiling for patients might be a promising approach for further investigation of these inhibitors in mCRPC and to identify reliable predictive biomarkers.

## 5. Conclusions

The oncological benefits of checkpoint inhibitors in mCRPC are unfortunately limited. Currently, there is no reliable predictive biomarker to suggest better response in patients. Future randomized trials based on genetic profiling of the patients could help to discover definitive biomarkers for patient selection before offering immunotherapy. 

## Figures and Tables

**Table 1:** Summary of the included studies. mCRPC, metastatic castration-resistant prostate cancer; ADT, androgen deprivation therapy; ORR, overall response rate; PSA, prostate-specific antigen; RECIST, response evaluation criteria in solid tumors; OS, overall survival; PD- L1, programmed death ligand 1.

Study	NCT ID/Trial Name	Phase and Status	Patient Criteria	Number of Patients	Drug	Primary Endpoint	Outcome
Antonarakis 2020 [21]	NCT02787005 (KEYNOTE-199)	Phase II, active, not recruiting	mCRPC with previous docetaxel and targeted ADT. Cohort1 (PD-L1-positive) Cohort 2 (PD-L1-negative). Cohort 3 (bone-predominant disease, regardless of PD-L1)	133 (cohort 1) 66 (cohort 2) 59 (cohort 3)	Pembrolizumab	ORR by RECIST 1.1	ORR was 5% (cohort 1) vs. 3% (cohort 2)
Sharma 2020 [19]	NCT02985957, (CheckMate 650)	Phase II, recruiting	mCRPC Cohort 1 (pre-chemo.), cohort 2 (post-chemo.)	45 (cohort 1) 45 (cohort 2)	Nivolumab + ipilimumab	ORR at 6 months, (rPFS) at 12 months	ORR—25% and 10%, median PFS—5.5 and 3.8 months in cohort 1 and 2, respectively
Sweeney 2020 [25]	NCT03016312 (IMbassador250)	Phase III, active, not recruiting	mCRPC after the failure of an androgen synthesis inhibitor and failure/ineligibility/refusal of a taxane regimen	759	Atezolizumab + enzalutamide vs. enzalutamide only	OS	Median OS 15.2 vs. 16.6 months, respectively
Hotte 2019 [26]	NCT02788773	Phase II, active, not recruiting	mCRPC after prior abiraterone and/or enzalutamide, and no more than one taxane	52	Durvalumab with or without tremelimumab	ORR measured by RECIST 1.1 and iRECIST	ORR 0% (0/13) vs. 16% (6/37) and PSA response rate 0% (0/13) vs. 16% (6/37) in the durvalumab arm vs. durvalumab + tremelimumab arm
Boudadi 2018 [20]	NCT02601014 (STARVE-PC)	Phase II, active not recruiting	mCRPC expressing AR-V7	15	Nivolumab + ipilimumab	Change in PSA response (>50% PSA decline)	PSA reponse-13.3% (2/15)
Karzai 2018 [22]	NCT02484404	Phase I/II Recruiting	mCRPC previously treated with enzalutamide and/or abiraterone	17	Durvalumab + olaparib	Improved PFS (70% PFS vs. an estimated 50% at 4 months)	rPFS of 51.5% at 12 months with a median rPFS of 16.1 months
Beer 2017 [23]	NCT01057810 (CA184-095)	Phase III, completed	Asymptomatic or minimally symptomatic with chemotherapy-naive mCRPC without visceral metastases	837	Ipilimumab vs. placebo	OS	Median OS 28.7 months vs. 29.7 months. No improvement in OS with ipilimumab
Kwon 2014 [24]	NCT00861614 (CA184-043)	Phase III, completed	mCRPC with progression after docetaxel	799	Ipilimumab vs. placebo after radiotherapy	OS	Median OS 11, 2 months vs. 10 months

**Table 2:** Newcastle–Ottawa Scale for risk of bias assessment of the non-randomized trials (scores ≥7–9, 4–6, <4 are considered as low, intermediate, and high risk, respectively).

Study	Selection				Comparability	Outcome			Overall
	Representativeness of Exposed Cohort	Selection of Not Exposed	Ascertainment of Exposure	Outcome Not Present at Start		Assessment of Outcome	Adequate Follow-up Length	Adequacy of Follow-up	
Antonarakis 2020 [21]	*		*	*	*	*	*	*	7/9
Sharma 2020 [19]	*		*	*	*	*	*	*	7/9
Hotte 2019 [26]	*			*	*		*	*	5/9
Boudadi 2018 [20]	*		*	*	*	*	*	*	7/9
Karzai 2018 [22]	*		*	*	*	*		*	6/9

* Each item is assessed by one star and the comparability by two stars (the total is nine stars).

## Data Availability

Not applicable.
